# Maternal Mortality in Nigeria: Holding the Line in Uncertain Times

**DOI:** 10.5334/aogh.4710

**Published:** 2025-03-25

**Authors:** Gabriel Dogbanya

**Affiliations:** 1 Doctoral student, Maternal and Child Health, Family Science Department, University of Maryland, College Park, Maryland, USA; 2 Fellow, West African College of Surgeons, Faculty of Obstetrics and Gynecology (FWACS), Lagos, Nigeria; 3 Fellow, National Postgraduate Medical College of Nigeria, Obstetrics and Gynecology (FMCOG), Lagos, Nigeria

**Keywords:** Maternal Mortality, Nigeria, Uncertain Times

Maternal mortality remains a nightmare in Nigeria. It highlights the adverse state of its health systems and indices among the committee of nations. It is a scourge to women in developing countries, particularly sub‑Saharan Africa. In an uncertain political climate in the United States and around the world that threatens the various programs and initiatives helping to fight and reduce the scourge, the Nigerian government must rise to the occasion and hold the line so it continues the modest gains it has been making in recent years in reducing maternal mortality. This article reviews maternal mortality in Nigeria in light of recent world events and the implications for donor‑dependent initiatives in developing countries aimed at tackling various health challenges.

The tragedy of maternal death has enormous consequences, not only for families but also for society in general [1]. For a country such as Nigeria (the most populous nation in Africa), any stoppage to the various initiatives aimed at tackling the challenges will be dire, with devastating consequences, in a country that is already reeling with poor health indices and is among an infamous trio with the world’s worst maternal death indices [2].

A global report revealed that Nigeria has the highest estimated maternal death rate, accounting for over one‑quarter (28.3%) of all estimated global maternal deaths, with approximately 8,200 maternal deaths [2] and a maternal mortality ratio of 1,047 per 100,000 livebirths [2]. With only 43% of births in the country assisted by a skilled provider, only 39% of births taking place in a health facility, and 59% delivered at home, [3] Nigeria has a lot of clinical and advocacy work to do to drastically reduce maternal mortality to meet the target of Sustainable Development Goal (SDG) 3 [4].

With the foreign aid funding freeze from major donor countries, such as the United States, Nigeria and indeed developing countries in Africa and Asia must think creatively outside the box to ensure life‑saving programs and interventions continue in their countries, until these periods of uncertainty clear. It provides a unique opportunity to look inward and come up with sustainability plans that are not dependent on donors. It will require bold, meticulous, practical, and locally acceptable strategies and programs at every level—modified on the basis of sociocultural peculiarities, challenges, and prevalent etiology in the various regions—to tackle the problems.

The inability to obtain high‑quality healthcare in most Nigerian health facilities contributes significantly to the high maternal mortality ratios. [5] Most Nigerian healthcare facilities, especially primary healthcare centers, are without skilled personnel, who instead are attracted to high‑paying urban centers [5].

Nigeria is making modest gains in the reduction of maternal mortality by taking strategic decisions, such as the “free emergency cesarean sections” (C‑section) policy, [6] which was introduced in late 2024 and was aimed at poor and vulnerable women, who were at high risk of dying due to the lack of access to cesarean sections. As the Health Minister said at the time, “no woman should lose her life simply because she can’t afford a C‑section.” It was a policy directed at reducing maternal mortality.

With the new realities, Nigerian officials have launched a committee to develop a transition and sustainability plan for US Agency for International Development (USAID)‑funded health programs, following US President Donald Trump’s 90‑day halt of most foreign aid [7]. The aim was to secure financial support to enable critical health programs to continue running. This is a commendable step, which all donor‑dependent countries should emulate. It is time to look inward and forward, hold the line, and ensure life‑saving healthcare services continue beyond this period of uncertainty, irrespective of the outcome.
